# Use of qualitative and mixed methods research in diagnostic technology evaluation and implementation: insights from a systematic review

**DOI:** 10.1186/s41512-026-00240-z

**Published:** 2026-10-07

**Authors:** Caity Roleston, Umasha Ukwatte, Nia Roberts, Gail N. Hayward, Philip J. Turner, Margaret Glogowska

**Affiliations:** 1 Nuffield Department of Primary Care Health Sciences, University of Oxford, Oxford, UK; 2 NIHR HealthTech Research Centre in Community Healthcare, University of Oxford, Oxford, UK; 3 Bodleian Health Care Libraries, University of Oxford, Oxford, UK

**Keywords:** Systematic review, Diagnostic test, Diagnostic technology, Evaluation, Qualitative, Mixed methods

## Abstract

**Background:**

Development of new diagnostic health technologies is increasing, alongside interest in how they can be implemented into health services. Evaluation of new diagnostic tests has typically consisted of technical and diagnostic accuracy, place in the clinical pathway, effectiveness and cost-effectiveness. However, there are other considerations which are important in determining their ultimate adoption. These include the usability and acceptability of new tests – how clinicians and patients respond to and interact with them – as well as how applicable they are to individual settings and contexts. Such issues are more likely to be explored and understood using qualitative and mixed methods research. The purpose of this systematic review was to discern in the literature which qualitative and mixed methods approaches have been used in evaluating and implementing diagnostic technologies and tests and to identify how future use of these methods can be supported in this rapidly developing field.

**Methods:**

We searched Medline, Embase and CINAHL from inception to September 2025 for evaluations of diagnostic technology and tests including qualitative and mixed methods research. The content and quality of the retrieved studies was examined using critical appraisal frameworks appropriate for the type of study, Spencer et al (2003) for qualitative studies and Mixed Methods Appraisal Tool (MMAT) (2018) for mixed methods studies.

**Results:**

Our search yielded 11331 results, with 28 studies included in the review. All the studies were evaluations of point of care technology or tests for diagnosis. The studies were relevant to seven different medical conditions, across 18 countries. Of the 28 studies, 18 used a qualitative research design only and 10 a mixed methods design. Most studies used semi-structured interviews with health care professionals as either the sole or principal data collection method. There were issues around quality and reporting, including engagement with the methodological literature especially with regard to data analysis, idiosyncratic use of terminology and guiding frameworks, and lack of exploration of patient perspectives.

**Conclusions:**

The review suggests that guidance to support the use of qualitative and mixed methods approaches in this field would be timely and helpful.

**Supplementary Information:**

The online version contains supplementary material available at https://doi.org/10.1186/s41512-026-00240-z.

## Introduction

The development of new types of diagnostic health technologies is accelerating and interest is growing in how they can be implemented in UK health services [1]. New tests need to be rigorously evaluated and in many parts of the world, considerable resources are already allotted to assessing and comparing emerging technologies and tests [2]. Typically, evaluation of new diagnostic tests considers a number of aspects, including the technical accuracy of the diagnostic test (its reliability in standardized conditions outside clinical practice); its place in the current clinical pathway; its diagnostic accuracy (its performance in confirming or excluding a target condition); how the test affects patient outcomes; and its cost-effectiveness [2]. Several different test evaluation frameworks have been proposed [3, 4], which acknowledge the need to explore impacts which go beyond clinical effectiveness and cost-effectiveness [5]. However, such broader impacts – social, psychological, organisational, ethical and legal – are ill-defined and there is a lack of clarity about how they should be evaluated.

In the UK, the National Institute for Health Care Excellence (NICE) Diagnostics Assessment Programme forms part of NICE’s activities in evaluation of medical technology, both undertaking and providing guidance for evaluation [6]. It acknowledges that outcomes for patients are dependent on the treatment which is based on the result of the diagnostic test and therefore, the value of the test or technology can be best understood in the context of its effect on the clinical care pathways. As it may not be obvious where in care pathways diagnostic technology is best placed, different options for placement within the pathway may end up being evaluated. As well as determining how new tests fit into existing clinical care pathways, there are other questions around diagnostic technologies and tests which are important and play a role in determining their adoption. These include their acceptability to clinicians and patients, practicalities surrounding their use, as well as broader consequences of testing which reach beyond clinical effectiveness and cost-effectiveness, such as clinical confidence in performing testing and interpreting results to achieve an accurate diagnosis, which can in turn lead to more rapid decision-making and reduce diagnostic misclassification for individual patients.

However, such issues and challenges cannot be fully explored or understood using quantitative designs alone. Qualitative methodologies (and by extension, mixed methods) are highly suitable for addressing broader questions about diagnostic tests mentioned above. Indeed, we have seen increasing use of qualitative methods in the development, piloting and evaluation of healthcare and public health interventions [7, 8]. In order to understand how this “wider impact” of diagnostic technology is being addressed [3], it is important to establish which methods are being employed and in what ways. The purpose of this systematic literature review was to search the diagnostic technology literature to highlight which qualitative and mixed methods approaches have been utilised in evaluation and implementation studies to date, appraise these approaches and to identify what learning they can offer, if any, to support the future use of qualitative and mixed methods research in this rapidly developing field.

## Methods

### Review design

The principles of a ‘knowledge support’ rather than a ‘decision support’ review applied [9], in that the literature retrieved was subject to methodological review, summarising and synthesizing methodological/methods aspects only. For this reason, the review was ineligible for registration with PROSPERO as no assessment of the value of any test/testing process and no outcomes of direct patient or clinical relevance were included.

### Search strategy

The search strategy was developed and tested in consultation with a specialist health sciences librarian with expertise in diagnostics research. Relevant databases for searching included Medline (OvidSP), Embase (OvidSP) and CINAHL (EBSCOHost). These were considered the most likely to identify appropriate papers in the biomedical and allied health-related literature. Database searches were conducted from inception to 26th September 2025. A supplementary file containing details of search terms used is available (see Additional File 1).

### Inclusion criteria

We applied the following inclusion criteria:

- Primary research studies related to any type of diagnostic health technology or diagnostic test.
- Studies where tests/technologies were being implemented or evaluated.
- Studies using qualitative and/or mixed methods approaches.
- English language publications only and freely available (limited resources).
- Papers from 2000 onwards (from this time, awareness of qualitative and mixed methods approaches has increased; the MRC Framework was initially published in 2000 [7] and the landmark first edition of Handbook of Mixed Methods in Social and Behavioral Research (Tashakkori & Teddlie, 2003) [10] was published in 2003).

### Exclusion criteria

- Primary stand-alone quantitative studies of aspects such as technical accuracy, diagnostic accuracy, clinical effectiveness and cost-effectiveness of diagnostic tests.
- Studies where screening (defined by the UK government as “the process of identifying apparently healthy people who may have an increased chance of a disease or condition” [11]) was the indication for testing.
- Focus on new diagnostic test implementation or evaluation for management/monitoring of long-term conditions.
- Studies reporting only quantitative evaluation, often using survey/questionnaire methods or other quantitative tools to explore patient/clinician/stakeholder views.
- Studies which were not focused on the evaluation or implementation of particular tests/technology but on wider research programmes of which the tests/tech were only a part
- Studies exploring general views of patient and/or clinicians about diagnostic tests/technology (outside implementation or evaluation).
- Studies exploring access to diagnostic tests for patients.
- Studies reporting clinicians’ knowledge of tests/clinician training programmes and education for tests.
- Studies exploring the current use and possible future use of diagnostic test/technology.
- Studies exploring barriers and facilitators generally, in advance of implementation and evaluation of tests/technology
- Studies reporting consensus around use of tests
- Studies discussing hypothetical views about the evaluation or implementation of a diagnostic test.
- Non-empirical articles such as book reviews, letters, opinions articles.
- Surveys e.g., of attitudes to diagnostic technologies, of current use of diagnostic technologies.
- Protocols.

### Screening, data extraction and appraisal

We developed and piloted guidance for screening. Three researchers (CR, MG, UU) used it to undertake title/abstract and full text screening, using Covidence (https://covidence.org) to record decisions and additional comments. Two reviewers were engaged in the initial screening but resources were not available for double-screening of all the papers. However, where there was any lack of clarity from details in the title, key word and abstract content of the papers, we checked the full paper. Where there was any uncertainty about the suitability of an individual paper, it was flagged for discussion and the decision to include or exclude was a joint one. All full text screening required consensus between two reviewers to include/exclude. Any discrepancies and uncertainties regarding eligibility were discussed and resolved as a team.

A data extraction form was developed to summarise the core characteristics of the included studies, such as study aims and setting; participant type/eligibility; condition/core features of diagnostic test; data collection method(s); data analysis method(s); and stakeholder engagement activity. Initial data extraction was conducted by UU and CR and iteratively reviewed and refined by UU, CR and MG.

The quality of the retrieved studies was also examined (by UU, CR and MG), to explore the rigour and appropriateness of the approaches used. Critical appraisal frameworks appropriate for the type of study, Spencer et al. (2003) [12] for qualitative studies and Mixed Methods Appraisal Tool (MMAT) (2018) [13] for mixed methods studies, modified to fit with the ‘knowledge support’ purpose of the review, were used. Studies were not excluded based on quality.

## Results

**Fig. 1:**
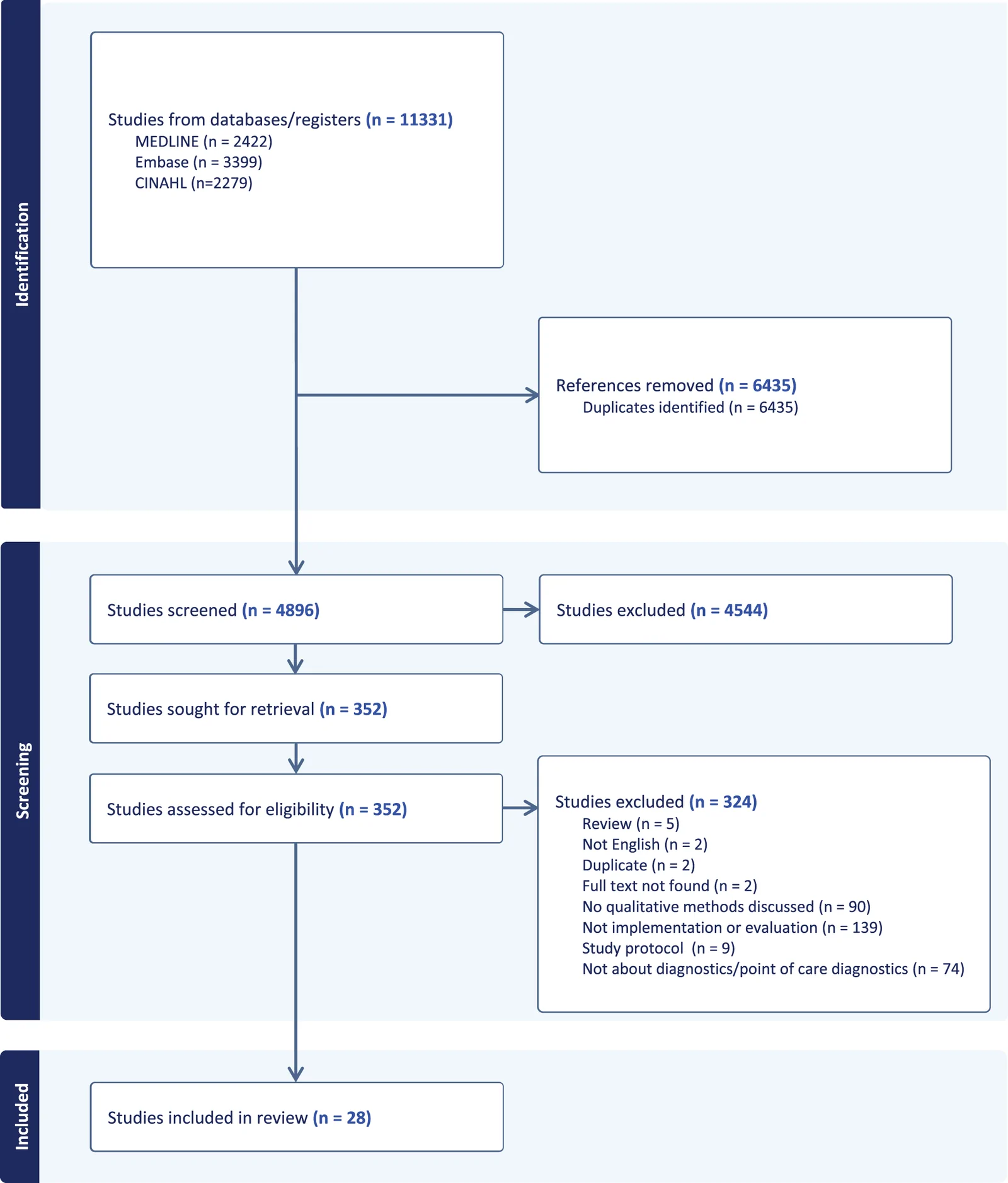
PRISMA diagram

Electronic searching of databases yielded 11,331 studies, with 6435 duplicates identified and removed, leaving a total of 4896 articles for title and abstract screening. 4544 studies were excluded for irrelevance and 352 full-text studies were assessed for eligibility. Of these, 324 were excluded, resulting in 28 studies being included in the systematic review. (See Fig. 1 PRISMA diagram for details).

### Characteristics of included studies

#### Setting

Of the 28 included studies, just over half (*n* = 15) were conducted either in the UK [14–21] or Europe [22–28]. Seven studies were conducted in Africa [29–35]. The remaining studies were conducted in North America [36–38]; Southeast Asia [39]; New Zealand [40]; and Australia [41]. (See Table 1 for Study Characteristics)

**Table 1:** Study characteristics

First Author, Year, Country	Study Aims	Setting	Condition; Diagnostic POCT (i.e., name of device, sampling technique)	Design; Conceptual Framework (if any)	Participant Eligibility (in Qualitative Component)	Data Collection (methods, number of participants, date)	Data Analysis	Public, Patient, or Stakeholder Engagement
Asiimwe, 2012, Uganda	To better understand the experiences of health care professionals using malaria rapid diagnostic tests (mRDTs); with a view to identify barriers and facilitators to implementation	Public healthcarecentres	Malaria; Malaria Rapid Diagnostic Test (mRDT) Pf immuno-chromatographic test (ICT) HRP2 type (histidine rich protein-2) mRDT ICT Patient capillary blood sample	Qualitative descriptive design Conceptual framework for technology acceptance and use was adapted for this study (Jeng, 2004)	Health care professionals who had one-month (minimum) experience with mRDTs and were involved in fever case management	Open-ended interviews (*n* = 102) conducted monthly starting in June 2007 (6–8 weeks after initial deployment of mRDTs)	Thematic analysis using realist method (no reference)	“Opinion leaders” provided feedback on the research
Bennis, 2024, Morocco	To investigate the micro-costing and acceptability of Cutaneous Leishmaniasis diagnostic tool	Primary Health Centres and District Laboratories	Cutaneous Leishmaniasis (CL); CL Detect Rapid Test (Inbios International Seattle, USA) Sample dental broach on lesion	Mixed methods design (micro-costing & acceptability)	Health care professionals who frequently managed CL patients at each site with experience using the CL-RDT or with some knowledge about the device as well as participants unaware about the CL-RDT	In-depth interviews (*n* = 46) conducted 26th August 2019–28 January 2020	Thematic analysis (Ziebland et al., 2006)	None
Blattner, 2019, New Zealand	To describe patient impact, health care professional acceptability, and cost-benefit analysis of POC haematology in hospital setting	Emergency and acute care	Broad indication; POC haematology benchtop analyser (Abbot CELL-DYN Emerald 18 analyser Abbott Diagnostics, Auckland, NZ)	Mixed methods design (cost analysis & acceptability)	All doctors, nurses and Taumata (Hokianga Health cultural advisors) working at site	Focus group discussion (*n* = 3) (first group with *n* = 7, second group *n* = 12); individual interview (*n* = 1) conducted between June 2016 and January 2017	Thematic analysis (no reference)	None
Boadu, 2016, Ghana	To identify the factors that influence implementation of mRDTs in Ghanian primary care	Primary healthcare facilities (included health centres, hospitals, and maternity homes)	Malaria; Malaria Rapid Diagnostic Test. (device/sampling technique not stated)	Qualitative descriptive design (ethnography) Conceptual framework for technology acceptance and use was adapted from Asiimwe (2012) for this study	Health care providers at participating facilities	Combined observation (120 hours); informal interviews (*n* = 21); Semi-structured interviews (*n* = 12); focus groups (*n* = 16 across 3 FGs) Conducted November 2011-October 2012	Thematic analysis (Roper & Shapira, 2000)	Site selection informed by study advisory group (community leaders and key informants) and local advisors were consulted during analysis
Boere, 2022, Netherlands	To determine intervention quality and barriers and facilitators to the implementation of C-Reactive Protein (CRP) POCT in nursing homes	Nursing homes	Lower respiratory tract infection (LRTI); C-Reactive Protein (CRP) POCT-analyzer Quikread go (Aidian Oy, Espoo, Finland). Patient capillary blood sample	Mixed methods process evaluation (UPCARE study)	Health care professionals at participating sites (purposive sampling informed by inclusion rate during the trial, ward size, and physician turnover)	Focus groups with physicians and nurses from 3 sites conducted in June and July 2010 (number of focus groups and participants not stated)	No named analytic approach (or reference) - open, axial, and selective coding mentioned	None
Brookes-Howell, 2019, International (Wales, England, Spain, Netherlands)	To explore the challenges clinicians face in diagnosing and managing urinary tract infection (UTI), and the extent to which a POCT can be used to inform their management decision(s)	Primary care	UTI; Flexicult POCT Patient urine sample	Qualitative descriptive design (POETIC trial)	Health care professionals working on the trial (purposive sampling informed by inclusion rate during the trial and range of experience using POCT across countries)	Semi-structured interviews (*n* = 35) conducted July-September 2015	Thematic analysis (Braun & Clarke, 2006)	None
Cals, 2010, Netherlands	To explore GP’ experiences of and attitudes towards CRP POCT for LRTI in primary care	Primary care	LRTI; CRP POCT test device (device brand/sampling technique not stated)	Qualitative descriptive design (IMPAC [3]T Study)	GPs from general practices in the trial	Semi-structured interviews (*n* = 20) conducted during first winter after patient recruitment (2008)	Thematic content analysis (Green & Thorogood, 2004)	Topic guide was piloted with GP outside of main study
Dahm, 2017, Australia	To understand health care professionals’ perspectives on the impact of a POCT rollout across rural and remote emergency departments	Emergency departments (rural and remote)	Variety of POCTs with broad indication for use of POCT (list of devices/sampling techniques not stated)	Qualitative descriptive design	Sites were selected purposively (informed by number of presentations per month and percentage of POCT usage). Health care professionals at participating sites were eligible if they had direct experience using POCT	Semi-structured interviews (*n* = 4)/ focus groups (*n* = 10) conducted August 2015 (14–15 months after initial implementation)	Thematic and content analysing using constant comparative method (Corbin & Strauss, 2014; Patton, 2015)	None
Daly, 2023, USA	To describe the characteristics of participating networks in a trial of the device and the complications challenging its execution.	Primary care practice-based research networks	SARS-CoV-2; 2322 Device Patient saliva sample	Qualitative descriptive design (2322 Trial)	Health care professionals from trial sites	Narrative semi-structured interviews (*n* = 9); not stated when interviews were conducted	Data analysis not described	None
De Vos, 2023, South Africa	To assess the usability and acceptability of POCT to detect gonorrhoea	Public primary healthcare facilities and community health centres	Neisseria Gonorrhoea (NG); NG-Lateral Flow Assay (LFT) and reader prototype POCT. Patient vaginal swab (for female patients) or first-void urine (for male patients)	Mixed-methods evaluation study Health Technology Adoption Framework (Ansbro et al., 2015; Asiimwe et al., 2012)	Health care workers either directly or indirectly involved with implementation	In-depth interviews (*n* = 21) conducted over four time-points (dates not stated)	Template analysis (no reference) deductive analysis based on Health Technology Adoption Framework	None
Dixon, 2021, UK	To explore clinicians’ perspectives and experiences using POCT in primary care (out-of-hours)	Primary care (out-of-hours)	POCT broad indication; Abbott i-stat machine with two panels of tests CHEM 8 and CG4. Patient venous blood sample	Qualitative descriptive design (part of mixed methods evaluation)	Clinicians who carried out out-of-hours home visits in the participating sites	Semi-structured interviews (*n* = 19) conducted November 2016-December 2017	Constant comparative method (Silverman, 2017)	None
Dixon, 2022, UK	To explore impact on prescribing of a POCT in primary care (out-of-hours) and clinician perspectives	Primary care (out-of-hours)	Respiratory tract infection (RTI); CRP POCT Abbott Afinion™ 2 Analyzer. Patient capillary blood sample	Mixed methods evaluation	Clinicians working at the three test sites	Semi-structured interviews (*n* = 18*) conducted January-December 2019 *One GP and one ANP were interviewed twice	Thematic analysis (Pope et al., 2000) using mind-mapping techniques (Ziebland & McPherson, 2006)	None
Eley, 2018, UK	To explore clinicians’ views of CRP POCT in primary care; with a view to identify barriers and facilitators to implementation	Primary care	LRTI; CRP POCT (brand not stated) Patient capillary blood sample	Qualitative descriptive design (part of mixed methods evaluation) COM-B Framework (Michie et al., 2011)	Clinicians at all trial sites (including control, intervention sites, and sites who rejected offer of CRP POCT) purposively recruited (informed by role and level of experience with CRP POCT)	Total (*n* = 26) via semi-structured interviews, focus group, or handwritten response conducted at the end of the trial (July 2017)	Thematic analysis (no reference) performed inductively followed by deductive mapping to COM-B framework (Michie et al., 2011)	Includes PPI statement (patients not involved in this study)
Geis, 2023, Switzerland	To explore general practitioners’ views on using procalcitonin and lung ultrasonography in primary care; with a view to identify barriers and facilitators to implementation	Primary care	LRTI; procalcitonin (PCT) POCT (device brand not stated) & lung ultrasonography PCT; Patient venous blood sample	Qualitative descriptive design (UltraPro Trial) Grounded theory method (Fasseur, 2018; Charmaz, 2006)	Pre-defined (criteria not stated) sample of GPs selected from trial	Semi-structured interviews (*n* = 12) conducted autumn-winter 2019–2020	Grounded theory method (Charmaz, 2006)	Includes PPI statement (patients not involved in this study)
Hayward, 2020, UK	To evaluate test usage and patient and clinician experience following the introduction of point of care (POC) blood tests into a primary care out-of-hours service	Primary care (out-of-hours)	POCT broad indication; Abbott i-STAT machine with two panels of tests CHEM 8 and CG4 Patient venous blood sample	Mixed methods service improvement evaluation	Clinicians who had access to POCT from study sites	Semi-structured interviews (*n* = 19); not stated when interviews were conducted	Thematic analysis (Strauss & Corbin, 1990)	Project protocol and patient facing materials reviewed by PPI group
Hodge, 2025, UK	To understand whether it is practical to use POCT in the pre-hospital setting for patients with lower acuity presentations	Pre-hospital	POCT broad indication; Abbott i-STAT machine with two panels of tests CHEM 8 and CG4 Patient venous blood sample	Sequential explanatory mixed methods feasibility study	Paramedics participating in feasibility study	Semi-structured interview focus group (*n* = 4); conducted after patient recruitment phase (date not specified)	Organised into themes (no reference); field notes only	None
Jung, 2025, Germany	To provide insights into CR POCT use in primary care	Primary care	Broad indication; CRP POCT (device brand not stated) Patient sample not stated	Qualitative descriptive observational design	Clinicians participating in observational study	Semi-structured interviews (*n* = 10); conducted May-August 2023	Content analysis (Kuckartz & Rädiker, 2022)	None
Kornelsen, 2023, Canada	To evaluate family physicians’ experience and use of POCUS in their clinical practice	Primary care	Broad indication; POCUS (device/brand not stated) Patient sample not stated	Qualitative descriptive design (IN PoCUS program) Diffusion of innovation framework (Rodgers, 1962)	Physicians enrolled on the IN PoCUS program	In-depth interviews (*n* = 21); not stated when interviews were conducted	Thematic analysis (Braun & Clarke, 2006)	None
Pines, 2018, USA	Quantitative study sought to evaluate impact of POCT on time of patient arrival to test result and length of stay Qualitative study sought to explore clinician views on effect of POCT on patient care; with a view to identify barriers to implementation	Community emergency department	POCT broad indication; Abbott POC i-STAT cardiac troponin (cTnI) and i-STAT Chem 8+ Patient venous blood sample	Mixed methods before and after evaluation	Clinicians working in the emergency department throughout implementation	Semi-structured interviews (*n* = 5) conducted in 2016	Grounded theory method (Glaser & Strauss, 1967)	None
Rutter, 2024, UK	To explore the experiences of health care professionals and patients on the use of FebriDx for lower respiratory tract infections	Primary care – General practice	LTRI; FebriDx Patient finger-prick blood	Qualitative descriptive design (Nested within the feasibility study of PREFIX Study)	Health care professionals with a role in administering and/or interpreting FebriDx testing, and/or communicating the results to patients as part of PREFIX Study Patients had undergone FebriDx testing for suspected LRTI	Semi-structured interviews with patients (*n* = 12) conducted February-June 2023, and Health care professionals (*n* = 10) conducted April-July 2023	Thematic analysis using an inductive, constant-comparative approach (Braun & Clarke, 2006; Thomas, 2006)	PPI group (*n* = 3) provided input into the study design, topic guides, data interpretation, and dissemination
Shahrzad, 2023, Denmark	To explore how a device for PCR (Cobas® Liat®) testing for respiratory tract infections was adopted and experienced by health care professionals in GP practice. And to identify facilitators and barriers for implementation	Primary care – General practice	SARS-COV-2 & Influenza A/B, Influenza A/B & RSV and Strep A; Cobas® Liat® PCR test (Roche Diagnostics) Patient sample not stated	Qualitative descriptive design	Health care professionals at study sites who performed test	Semi-structured interviews with health care professionals post-implementation (*n* = 11) and pre-implementation (*n* = 4) conducted March-June 2022 across 11 sites	Analytic method not stated Coding involved identifying use of technology and barriers and facilitators to implementation	None
Simonsen, 2024, Denmark	To explore the experiences of health care professionals on the use of POC PCR for respiratory tract infection	Primary care – general practice	SARS-COV-2 & Influenza A/B, Influenza A/B & RSV and Strep A; POCT name not stated (manufactured by Roche Diagnostics) Patient swab sample (unspecified)	Qualitative ethnographic study (part of feasibility study) Rosa’s (2010) social theory of technological acceleration (concepts of alienation & resonance)	Observations of all consultations (even though not related to POC PCR) Eligibility of semi-structured interviews not stated	2–3 full day observations of all consultations in 4/5 included sites; semi-structured interviews (*n* = 14). Date(s) conducted not stated	Analytic method not stated Coding involved identifying barriers and facilitators to implementation	None
Suttels, 2023, Benin	To gain in-depth insights into clinicians’ views on point of care lung ultrasound (POC-LUS) and computer-assisted diagnosis (CAD) for the diagnosis of LRTI	National teaching hospital for Pneumology and Tuberculosis	LRTI; One probe point of care lung ultrasound (POC-LUS) (device/brand not stated)	Qualitative descriptive design (TrUST Study)	Treating physicians active in the study site	Semi-structured interviews (*n* = 8) conducted in September 2021	Grounded theory method (Fasseur, 2018; Morse et al., 2009)	Includes PPI statement (patients not involved in this study)
Tan, 2017, Singapore	To explore patient and health care professionals’ experiences of POCT to diagnose dengue fever	Hospital (centre for infectious diseases)	Dengue fever (virus NS1 antigen and antibodies); SD BIOLINE Dengue Duo rapid diagnostic POCT. Patient venous blood sample	Qualitative before and after evaluation study Process mapping (a patient-centred approach to evaluation) (Trebble et al., 2010)	Patients treated at the hospital before and after introduction of POCT Health care professionals working at the hospital site (some before some after POCT introduction); purposive sampling (informed by patient care journey)	Focus groups with patients (*n* = 21). Two focus groups were conducted before POCT (June 2013) and two after (date not stated) Semi-structured interviews with health care professionals (*n* = 20); not stated when interviews were conducted	Framework approach (Ritchie & Lewis, 2014)	None
Van den Bruel, 2016, UK	To assess the feasibility and acceptability of CRP POCT in out-of-hours general practice	Primary care (out-of-hours)	Acute RTI; CRP POCT Patient capillary blood sample	Mixed methods observational cohort with a nested randomised controlled trial and embedded qualitative study	Parent/guardian participating in the trial. Purposively sampled (informed by ethnicity, family composition, parent and child age, and randomisation) All clinicians were eligible.	Semi-structured interviews with parent/guardian (*n* = 12) (conducted within one month of inclusion, no date stated) Semi-structured interviews with health care professionals (*n* = 7) (conducted at study end, date not stated)	Thematic analysis (no reference)	Codes and themes were verified by public advisory panel
Williams, 2018, Tanzania	To evaluate the impact of malaria RDT on prescribing behaviour, assess patients’ and prescribers’ perceptions, and identify barriers to implementation	Rural public dispensaries	Malaria; Paracheck Pf (manufactured by Orchid Biomedical Systems (India)) Patient blood sample	Mixed methods pilot evaluation	Patients of facility (first 20–30 referred per dispensary) were invited for interview All providers who used the RDT were invited for interview All facility staff involved in any aspect of using RDT were invited for informal discussions	Patient interviews (*n* = 355); provider interviews (*n* = 21); informal discussions (*n* = not stated) Two evaluation weeks (four and eight of pilot implementation period)	Content analysis techniques (no reference)	None
Xie, 2024, UK	To explore factors that influence adoption or non-adoption of POCT for RTI in general practice	Primary care – general practice	Influenza A/B, RSV, and SARS-CoV-2; Cobas Liat PCR system Patient nasopharyngeal swab sample	Qualitative ethnographic study (nested within PIAMS POCT implementation study) Non-adoption, Abandonment, Scale-up, Spread, and Sustainability (NASSS) Framework (Not described as MM but questionnaire also conducted & reported)	Opportunistic sampling to select sites for observation with several months of accumulated experience with the POCT	Observations conducted in 4/10 study sites; conducted May 2023	Graphic notation technique (Business Process Modelling Notation) to visualise POCT swabbing workflow alongside developing Unified Modelling Language use-case diagrams	None
Zongo, 2018, Burkina Faso	To explore health care professionals’ experience of using RDT for malaria in Burkina Faso	Health and social promotion centres	Dengue fever; SD Bioline Dengue NS1, IgG/IgM (PanBio®, Seoul, South Korea) RDT Patient blood sample	Qualitative descriptive design	Health care professionals from participating health centres (both trained and untrained professionals were eligible)	Semi-structured interviews (*n* = 32) conducted May-June 2014	Content analysis (no reference)	None

Over half (*n* = 17) were conducted in primary care/general practice/pre-hospital care [14–21, 23–28, 31, 32, 37]. Health centres [29, 30, 35] and emergency care [38, 40, 41] were also frequently utilised settings. Single studies were conducted in a hospital [39]; nursing homes [22]; a practice-based research network [36]; a tertiary centre [33]; and a public dispensary [34].

#### Diagnostic test/technology features

All the included studies utilised point of care tests (POCTs). The POCTs were used to diagnose a variety of medical conditions, the most frequently implemented were for respiratory tract infections (*n* = 12) [14, 16, 19–22, 24–28, 33]. Other conditions included malaria (*n* = 3); [29, 31, 34] dengue (*n* = 2); [35, 39] SARS-CoV-2 (*n* = 1); [36] cutaneous leishmaniasis (*n* = 1); [30] gonorrhoea (*n* = 1); [32] and urinary tract infection (*n* = 1) [23]. In addition, seven studies investigated POCTs or Point of care ultrasound (POCUS) as part of general investigations to support diagnosis of a range of conditions with non-specific symptoms [15, 17, 18, 37, 38, 40, 41].

Over half (*n* = 16) of POCTs used required blood samples [14–20, 22, 24, 26, 29, 34, 35, 38–40]. POCUS images (*n* = 2); [33, 37] urine (*n* = 2); [23, 32] dental broach on an ulcerated skin lesion (*n* = 1); [30] saliva (*n* = 1), [36] nasopharyngeal and vaginal swab (*n* = 1) samples were also used [21, 32]. One study combined procalcitonin POCT (sampling technique unspecified) and POCUS [25]; one study used an unspecified swabbing technique [28]; and three studies do not describe POCT sample requirements or sampling techniques [27, 31, 41].

#### Context

In terms of context, the qualitative studies (*n* = 18) were standalone explorations of health care professional (HCP) experience using particular diagnostic tests [15, 16, 19, 23–26, 28, 29, 37, 41], of HCP and patient experiences of the introduction of a test [39], and of factors influencing the implementation of particular tests [21, 27, 31, 33, 35, 36]. Among the mixed methods studies (*n* = 10), two are described as evaluations of a service improvement [14, 17], one as a micro-costing and acceptability of a diagnostic tool [30], one as a feasibility study [18], one as a study of impact and acceptability of a test [40], one as a process evaluation of implementation of a test [22], one as a usability and acceptability study of a test [32], one as an evaluation of the feasibility and acceptability of a test [20], one as a pilot evaluation of a test [34], and one as an evaluation of a test on care and clinician views [38].

#### Patient/Public/Stakeholder involvement and engagement (PPIE)

Of the included studies, 19 do not mention involving the public/patients or other stakeholders in the development of their studies, and three studies confirm patients were not involved in the study [16, 25, 33]. Of the six which did involve patients/the public, two engaged a PPIE group to inform project design and materials [17, 19], and to review interpretation and dissemination [19]. Two studies consulted with relevant stakeholders (GPs and local public opinion leaders, respectively) to provide feedback on the proposed research [24, 29], and two studies involved patients/community leaders to verify their analysis [20, 31].

### Methods of included studies

Eighteen of the included studies employed only qualitative methods [15, 16, 19, 21, 23–29, 31, 33, 35–37, 39, 41] and ten a mixed methods design [14, 17, 18, 20, 22, 30, 32, 34, 38, 40]. One of the included studies is framed as a ‘qualitative implementation evaluation’ but combined qualitative with quantitative methods (questionnaire alongside ethnography). [21] For ease, we have included it as a qualitative study throughout the Results section presented here.

All but three of the included studies featured semi-structured interviews, typically as the sole qualitative method, but also in combination with focus groups [16, 31, 39–41] or observation/field notes [28, 31, 34].

For the majority of studies (*n* = 23), participants were healthcare professionals only [14–16, 18, 21–33, 35–38, 40, 41]. Five studies included both patients and healthcare professionals [17, 19, 20, 34, 39].

Just under half (*n* = 13) of the included studies used a thematic analysis to analyse the qualitative data [14–20, 23, 24, 29, 31, 37, 41]. While less frequently applied, other analytic approaches included Grounded Theory [25, 33, 38]; constant comparison [32]; Content Analysis [26, 34]; and a Framework Approach [39]. However, the analytic approach was not stated/otherwise unclear in four of the included studies [22, 27, 28, 36].

### Quality appraisal of included studies

An adapted version of Spencer et al.’s (2003) framework for evaluating the quality of qualitative evaluations [12] was applied to all the included studies. The ten mixed methods studies were also appraised using an abridged version of the MMAT, 2018 [13]. The qualitative appraisal tool was chosen as it was designed to be used in evaluations which suited the purpose of this review; the MMAT because it is was designed for use in mixed methods studies including different types of quantitative and qualitative designs, with a focus on integration. See Tables 2 and 3 for the quality appraisals of qualitative and mixed methods studies respectively.

**Table 2:** Quality appraisal for qualitative studies

	**Study Design**^**i**^	**Sample**^**ii, iii**^	**Data Collection**^**iv**^	**Data Analysis**^**v**^	**Reporting**^**vi, vii**^	**Ethics**^**viii**^
Asiimwe (2012)	Implicit justification – study aims align with qualitative design but no explicit rationale.	Sampling/recruitment strategy unclear. Recruitment goal not stated. Profile of participants (gender, job role etc.) not described. Number of non-participants stated, and reasons provided.	Who conducted data collection and aspects relating to practical arrangement described. Interviews not recorded; note making processes not described. Topic guide not described; not available as supplementary file.	Who performed analysis not stated. Thematic analysis using realist method – no reference – steps summarised. Initial inductive coding followed by deductive analysis using theoretical framework.	Greater attention is afforded the clinical observation results (which were numerical) and qualitative findings presented as secondary - often without participant quotations. Figure depicts key barriers and facilitators to implementation. Reporting guidelines not used.	Authors confirm ethical approval and process for informed consent. No attention to the influence of researcher positionality/ decisions on outcomes.
Boadu (2016)	Explicit justification.	Purposive selection of sites (criteria described). Sampling/recruitment for participants unclear. Participant profile described. Reasons for non-participation/no response not stated.	Who conducted data collection not stated; aspects relating to practical arrangement described. Topic guide described; not available as supplementary file.	Who performed analysis not stated. Thematic analysis (Roper & Shapira, 2000) – steps not described.	Presentation of analysis retains contextual information & is supported by appropriate participant quotes. Reporting guidelines not used.	Authors confirm ethical approval and process for informed consent. No attention to the influence of researcher positionality/ decisions on outcomes.
Brookes-Howell (2019)	Implicit justification – study aims align with qualitative design but no explicit rationale.	Combination of purposeful and whole population sampling. No rationale for sampling targets. Target of 10 interviews only achieved in one of four networks. Reasons for non-participation/no response not stated. Final sample included range of health care professionals.	Who conducted data collection and aspects relating to practical arrangement described. Interview topics/questions included in table.	Who performed analysis not stated. Thematic analysis (Bruan & Clarke, 2006) - several terms used are inconsistent with TA approach.	Presentation of analysis retains contextual information & is supported by appropriate participant quotes. Attention given to difference/ comparisons. Written up in an ‘applied’ way – speaking to topic guide sections rather than ‘themes.’ Reporting guidelines not used.	Authors confirm ethical approval and process for informed consent. No attention to the influence of researcher positionality/ decisions on outcomes.
Cals (2010)	Explicit justification.	Whole population sampling approach – all 20 participated. 10 general practices. Profile of participants described and compared to population profile.	Who conducted data collection and aspects relating to practical arrangement described. Interview topics/questions described; not included as supplementary file.	Who performed analysis stated. Thematic content analysis (Green & Thorogood, 2004) - steps for analysis listed but not described. Intercoder reliability stated (70%), and how disagreements handled.	Presentation of analysis retains contextual information & is supported by appropriate participant quotes. Attention given to difference/ comparisons. Reporting guidelines not used.	Confirms ethics for trial, but separate ethics for this study not required. No attention to the influence of researcher positionality/ decisions on outcomes.
Dahm (2017)	Justification provided for why semi-structured interviews were used; no justification for the use of focus groups.	Purposive sampling (criteria described). Recruitment goal not stated. Reasons for non-participation/no response not stated. Diverse sample achieved.	Who conducted data collection and aspects relating to practical arrangement described. Interview topics/questions described (not for focus groups); not included as supplementary file.	Who performed analysis stated. Constant comparative method as well as thematic and content analysis applied (Corbin & Strauss, 2014; Patton, 2015) - steps summarised.	Presentation of analysis retains contextual information & is supported by appropriate participant quotes. Reporting guidelines not used.	Authors confirm ethical approval and process for informed consent. No attention to the influence of researcher positionality/ decisions on outcomes.
Daly (2023)	Implicit justification – study aims align with qualitative design but no explicit rationale.	Sampling/ recruitment strategy unclear. Participant profile unclear – including how many people were included. Reasons for non-participation/no response not stated.	Authors do not describe who performed data collection. Interview topics/questions described; not included as supplementary file. Pertinent data collection details are included in data analysis section.	Analytic approach not described.	Table summarises challenges, consequences, and solutions to various aspects of trial. These are lists/descriptions with no obvious relationship to the data. There are no participant quotes. Reporting guidelines not used.	Authors confirm ethical approval and process for informed consent. No attention to the influence of researcher positionality/ decisions on outcomes.
Dixon (2021)	Explicit justification.	Purposive sampling strategy to gain perspectives from range of health care professionals for whom POCT were available. Recruitment goal not stated. Final sample included range of health care professionals and users & non-users of POCT. Reasons for non-participation/no response not stated.	Who conducted data collection and aspects relating to practical arrangement described. Interview topics/questions not described or included as supplementary file.	Who performed data analysis stated. Constant comparative method – no reference – steps summarised.	Presentation of analysis retains contextual information & supported by appropriate participant quotes. Spectrum of opinions presented & depth of data and analysis showcased. Reporting guidelines not used.	Authors confirm ethical approval and process for informed consent. No attention to the influence of researcher positionality/ decisions on outcomes.
Eley (2018)	Implicit justification – study aims align with qualitative design but no explicit rationale. Qualitative methods (focus groups & interviews) treated as equivalent.	All practices were approached until data saturation achieved. Purposive sampling (criteria described). Reasons for non-participation/no response not stated. Diverse sample achieved.	Who conducted data collection and aspects relating to practical arrangement described. Interview topics/questions described; not included as supplementary file.	Who performed data analysis stated. Thematic analysis – no reference – analytic steps poorly described.	Presentation of analysis retains contextual information & supported by appropriate participant quotes. Attention given to difference/ comparisons. Authors do not comment on themes/codes identified that did not easily map onto the framework. Reporting guidelines not used.	Authors confirm ethical approval and process for informed consent. No attention to the influence of researcher positionality/ decisions on outcomes.
Geis (2023)	Implicit justification – study aims align with qualitative design but no explicit rationale. Design based on theoretical and methodological principles of GTM; reference but no rationale.	A predefined sample of 12 GPs were included in the study (from 60 GPs in overall trial) - no explanation as to who/how these 12 were selected. Justification for sample size provided (saturation). All invited participated so no non-participants. Sample and study cohort do not statistically differ to each other on any of the recorded variables.	Who conducted data collection and aspects relating to practical arrangement described. Interview topics/questions described; not included as supplementary file.	Grounded Theory (Charmaz, 2006) but no rationale for methodology. Analysis performed independently by two researchers applying open, axial, selective coding. Mentions results were checked for intercoder reliability but does not report rate of agreement. Analysts recorded memos to explore their reflexivity, feelings and knowledge of the data (Fasseur, 2018 - relates to GTM).	Presentation of analysis retains contextual information & supported by appropriate participant quotes. Reporting guidelines not used.	Authors confirm ethical approval and process for informed consent. No attention to the influence of researcher positionality/ decisions on outcomes.
Jung (2025)	Implicit justification – study aims align with qualitative design but no explicit rationale.	Sampled from clinicians participating in observational study. Recruitment goal not stated. Participant profile described. No refusals/drop outs.	Who conducted data collection stated; aspects relating to practical arrangement described. Interview topics/questions developed using the updated Consolidated Framework for Implementation Research (CFIR) (referenced, not described); interview guide included as supplementary file.	Who performed data analysis stated. Deductive content analysis (Kuckartz & Rädiker, 2022) using CFIR. Reports intra-coder and inter-coder reliability. Levels of inter-coder (kappa = 0.88) and intra-coder reliability (0.96), which authors reported as ‘valid interpretation’.	Presentation of analysis retains contextual information & supported by appropriate participant quotes. No attention given to difference/ comparisons. Authors do not comment on themes/codes identified that did not easily map onto the framework. COREQ criteria for reporting qualitative research.	Authors confirm ethical approval and process for informed consent. No attention to the influence of researcher positionality/ decisions on outcomes.
Kornelsen (2023)	Explicit justification.	Whole population sampling approach. Recruitment goal not stated. Reasons for non-participation/no response not stated. Participants not described.	Who conducted data collection not stated; aspects relating to practical arrangement described. Interview topics/questions described and included as supplementary file.	Who performed data analysis stated. Thematic analysis (Braun & Clarke, 2006) analytic process described. Preliminary results shared with rural physicians & rural physician organisations for comments – high congruence. Analysis mapped to theory; process not sufficiently described.	Presentation of analysis retains contextual information & supported by appropriate participant quotes. Authors do not comment on themes/codes identified that did not easily map onto the framework. Reporting guidelines not used.	Authors confirm ethical approval and process for informed consent. No attention to the influence of researcher positionality/ decisions on outcomes.
Rutter (2024)	Implicit justification – study aims align with qualitative design but no explicit rationale.	Combination of purposeful (criteria stated) and snowball sampling. Recruitment goal not stated. Reasons for non-participation/no response not stated. Participant profile described; sample diversity achieved (excepting ethnicity).	Who conducted data collection stated; aspects relating to practical arrangement described. Interview topics/questions described and included as supplementary file.	Who performed data analysis stated. Thematic analysis (Braun & Clarke, 2006) analytic process described. PPI consultation for interpretations.	Presentation of analysis retains contextual information & supported by appropriate participant quotes. Attention given to difference/ comparisons. Standards for reporting qualitative research: A synthesis of recommendations (O’Brien et al., 2014).	Authors confirm ethical approval and process for informed consent. No attention to the influence of researcher positionality/ decisions on outcomes.
Shahrzad (2023)	Implicit justification – study aims align with qualitative design but no explicit rationale.	Eligibility, sampling, and recruitment strategies unclear. Recruitment goal not stated. Pre- and post-implementation interviews, but unclear if same participants were interviewed. Unclear how many sites conducted pre/post-implementation interview (non-participation due to time limitations); unclear which sites were/were not included in recruitment	Who conducted data collection stated; aspects relating to practical arrangement described. Interview topics/questions described (map onto Normalisation Process Theory) and included as supplementary file.	Who performed data analysis stated. Analytic approach not described but motivated to identify how test was used and barriers & facilitators to implementation.	Presentation of analysis retains contextual information & supported by appropriate participant quotes. Attention given to difference/ comparisons pre-post- implementation. COREQ criteria for reporting qualitative research.	Authors confirm ethical approval and process for informed consent. No attention to the influence of researcher positionality/ decisions on outcomes.
Simonsen (2024)	Implicit justification – study aims align with qualitative design but no explicit rationale.	Whole population sampling approach to ethnographic observation - all but one participated (reason given). Sampling, and recruitment strategies for semi-structured interviews not stated. Recruitment goal not stated. Profile of sites/participants not stated.	Who conducted data collection stated. Process for observation notes/memos not stated. Interview topics/questions described; not included as supplementary file.	Who conducted data analysis stated. Analytic approach not stated; analytic steps described. Initial coding identified barriers & facilitators which were mapped onto theoretical framework.	Presentation of analysis retains contextual information & supported by appropriate participant quotes. Attention given to difference/change over time. Reporting guidelines not used.	Authors confirm ethical approval and process for informed consent. No attention to the influence of researcher positionality/ decisions on outcomes.
Suttels (2023)	Implicit justification – study aims align with qualitative design but no explicit rationale.	Whole population sampling approach – all but one participated (reason given). Single urban practice. Participant profile described.	Who conducted data collection and aspects relating to practical arrangement described. Interview topics/questions described and included as supplementary file.	Grounded theory method - no reference or rationale. Uses appropriate terms (open, axial, selective coding) & coding was performed inductively by two analysts independently. Mentions results were checked for intercoder reliability but does not report rate of agreement.	Presentation of analysis retains contextual information & supported by appropriate participant quotes. Standards for reporting qualitative research: A synthesis of recommendations (O’Brien et al., 2014).	Authors confirm ethical approval and process for informed consent. No attention to the influence of researcher positionality/ decisions on outcomes.
Tan (2017)	Implicit justification – study aims align with qualitative design but no explicit rationale.	Maximum variation (criteria described); snowball sampling. Recruitment goal not stated. Reasons for non-participation/no response not stated. Participant profile described.	Who conducted data collection and aspects relating to practical arrangement described. Interview topics/questions described; not included as supplementary file.	Who performed data analysis stated. Framework approach (Ritchie & Lewis, 2014) analytic process described. Deductive analyses informed by framework.	Presentation of analysis retains contextual information & supported by appropriate participant quotes. Authors do not comment on themes/codes identified that did not easily map onto the framework. COREQ criteria for reporting qualitative research.	Authors confirm ethical approval and process for informed consent. No attention to the influence of researcher positionality/ decisions on outcomes.
Xie (2024)	Explicit justification.	Opportunistic sampling for ethnographic observation. Recruitment goal not stated. Reasons for non-participation/no response not stated. Site profiles described.	Who conducted data collection not stated. Collection of field memos & ad hoc informal interviews described. Interview topics/questions described; not included as supplementary file. (Questionnaire data also collected but not described as MM)	Who performed data analysis not stated. Process analysis combining Business Process Modelling Notation (Dumas et al., 2018) and Unified Modelling Language (Dobing & Parsons, 2000) analytic process not described.	Findings related to ethnographic observations centre around the development of a process flowchart and use-case diagram. Attention given to difference between health care professionals. Findings organised using NASSS domains (the technology, adopters, organisation) but analytic process not described in Methods. Standards for reporting qualitative research: A synthesis of recommendations (O’Brien et al., 2014).	Authors confirm ethical approval and process for informed consent. No attention to the influence of researcher positionality/ decisions on outcomes.
Zongo (2018)	Implicit justification – study aims align with qualitative design but no explicit rationale.	Goal to recruit from each of the participating sites - no specific numerical target mentioned. Purposeful sampling (criteria not stated). Included both people who had received training and those who had not to enable comparisons. Reasons for non-participation/no response not stated.	Who conducted data collection and aspects relating to practical arrangement described. Interview topics/questions described; not included as supplementary file.	Who performed data analysis not stated. Data analytic strategy not described.	Presentation of analysis retains contextual information & supported by appropriate participant quotes. Attention given to difference/ comparisons. Reporting guidelines not used.	Authors confirm ethical approval and process for informed consent. No attention to the influence of researcher positionality/ decisions on outcomes.

^i^How defensible is the research design?

^ii^How well defended is the sample design/target selection of cases/documents?

^iii^Sample composition/case inclusion – how well is the eventual coverage described?

^iv^How well was the data collection carried out?

^v^How well has the approach to, and formulation of, the analysis been conveyed?

^vi^How clear are the links between data, interpretation and conclusions – i.e. how well can the route to any conclusions be seen?

^vii^How clear and coherent is the reporting?

^viii^What evidence is there of attention to ethical issues?

**Table 3:** Quality appraisal for mixed methods studies

	**Study Design (Qualitative Component)**^**i**^	**Sample (Qualitative Component)**^**ii, iii**^	**Data Collection (Qualitative Component)**^**iv**^	**Data Analysis (Qualitative Component)**^**v**^	**Reporting (Qualitative Component)**^**vi, vii**^	**Ethics**^**viii**^	Rationale for Using Mixed Methods Design/Design Used	Integration of methods
Bennis et al. (2024)	Implicit justification – study aims align with qualitative design but no explicit rationale.	Stratified sampling of sites (criteria described). Whole population sampling approach (all health care professionals at sample sites invited). Recruitment goal not stated. Profile of participants (gender, job role etc.) described. Reasons for non-participation/no response not stated.	Who conducted data collection and aspects relating to practical arrangement described. Topic guides described; available as supplementary file.	Who performed analysis at each stage stated. Thematic analysis - analytic steps briefly summarised, including implementation of ‘one sheet of paper’ approach (Ziebland & McPherson, 2006).	Presentation of analysis retains contextual information & supported by appropriate participant quotes. Attention given to difference/ comparisons. Table summarises identified themes, but these are not reported in the text. Standards for Reporting Qualitative Research (SRQR) (O’Brien et al., 2014).	Authors confirm ethical approval and process for informed consent. No attention to the influence of researcher positionality/ decisions on outcomes.	Explicit rationale provided for using mixed methods - aims of quantitative and qualitative delineated. Design described as explanatory.	Authors use the qualitative interview findings to provide confirmation of some of the quantitative results.
Blattner et al. (2019)	Implicit justification – study aims align with qualitative design but no explicit rationale.	Whole population sampling approach (all health care professionals at sample sites invited). Recruitment goal not stated. Profile of participants (gender, job role etc.) not described. Reasons for non-participation/no response stated.	Who conducted data collection and aspects relating to practical arrangement described. Topic guides described; not available as supplementary file.	Who performed analysis not stated. Thematic analysis (no reference) - analytic stages not described. Analysis combined inductive & deductive logic.	Presentation of analysis retains contextual information & supported by appropriate participant quotes. Reporting guidelines not used.	Authors confirm ethical approval and process for informed consent. No attention to the influence of researcher positionality/ decisions on outcomes.	Explicit rationale provided for using mixed methods - aims of quantitative and qualitative delineated. Concurrent design – stated explicitly.	Integration during interpretation – stated explicitly in Methods. However, Results of each method are presented separately with no integration. No explicit integration in Discussion.
Boere et al. (2022)	Implicit justification – study aims align with qualitative design but no explicit rationale.	Purposive selection of sites (criteria described). Recruitment goal not stated. Profile of participants (gender, job role etc.) not described. Reasons for non-participation/no response not stated.	Who conducted data collection and aspects relating to practical arrangement not described. Topic guide described; not available as supplementary file.	Who performed analysis at each stage stated. No named analytic method (no references).	Presentation of analysis retains contextual information & supported by appropriate participant quotes. Cannot tell data collected via focus group. Lack of headings/sub-headings undermine readability. Reporting guidelines not used.	Authors confirm ethical approval and process for informed consent. No attention to the influence of researcher positionality/ decisions on outcomes.	No rationale provided for mixed methods design.	No integration - findings and discussion attend to each in turn, no links are made between the two.
De Vos et al. (2023)	Implicit justification – study aims align with qualitative design but no explicit rationale. Study was time series, multiple data collection points - rationale inferred but not stated explicitly.	Combination of whole population and purposive sampling (criteria not stated). Recruitment goal not stated. Participant profile described. Reasons for non-participation/no response not stated.	Who conducted data collection and aspects relating to practical arrangement described. Interview topics described; not available as supplementary file.	Who performed analysis stated. Constant comparison (no reference) approach guided by framework. Brief summary of analytic steps.	Presentation of analysis retains contextual information & supported by appropriate participant quotes. Attention given to difference/ comparisons. Authors attend to the time-based aspect of analysis by presenting findings chronologically - this is effective at observing change over time. No reporting guidelines used.	Confirms ethics approval was granted, and participants consented. No attention to the influence of researcher positionality/ decisions on outcomes.	No rationale provided for mixed methods design. Embedded design but not explicitly stated.	No integration - findings and discussion attend to each in turn, no links are made between the two.
Dixon et al. (2022)	Implicit justification – study aims align with qualitative design but no explicit rationale.	Whole population and purposive sampling (criteria stated). Recruitment goal not stated. Sample drawn from clinicians from all involved sites. Non-responders/non-participants not addressed. Type of clinician role described but other sample characteristics not included.	Who conducted data collection and aspects relating to practical arrangement described. Interview topics described; available as supplementary file.	Who performed analysis stated. Thematic analysis (Pope et al., 2000) using mapping technique (Ziebland & McPherson, 2006) - steps for analysis listed but not described.	Presentation of analysis retains contextual information & supported by appropriate participant quotes. No reporting guidelines used.	Ethics approval confirmed and mention of consent for qualitative component. No attention to the influence of researcher positionality/ decisions on outcomes.	No rationale provided for mixed methods design. Described as a concurrent approach where quantitative and qualitative components proceeded in parallel through the same study period, were analysed separately and integrated.	The integration is explicitly included in the Discussion, with the qualitative findings being used to explain the quantitative findings.
Hayward et al. (2020)	Implicit justification – study aims align with qualitative design but no explicit rationale.	Whole population (clinicians in study sites who had used POCT) presumed. GPs interviewed twice (start and end) but no rationale given. Brief profile of participants (job role, years in role etc.). Non-responders/non-participants not addressed for clinicians. Authors explain reasons for low interview uptake among patients.	Who conducted data collection and aspects relating to practical arrangement described. Topic guide not described; not available as supplementary file.	Who performed analysis not stated. Analysed using thematic analysis (Strauss & Corbin, 1990) – analytic stages not described.	Presentation of analysis retains contextual information & supported by appropriate participant quotes (although sub-themes are very brief). Attention given to differences observed in differences in testing between sites. No reporting guidelines used.	Ethics approval confirmed and mention of consent for qualitative component. No attention to the influence of researcher positionality/ decisions on outcomes.	Explicit rationale provided for using mixed methods - aims of quantitative and qualitative delineated. Embedded design – explicitly stated.	Authors leverage the qualitative findings to provide insight into the poor uptake of the intervention. Authors do integrate qualitative and quantitative findings in discussion that demonstrates added value - but this is not stated explicitly.
Hodge et al. (2025)	Implicit justification – study aims align with qualitative design but no explicit rationale.	Whole population sampling approach (all paramedics participating in feasibility invited). Recruitment goal not stated. All paramedics still engaged with study participated in focus group. Profile of participants (gender, job role etc.) described. Reasons for non-participation/no response not stated.	Unclear who or where focus group conducted; duration of focus group stated. Topics not described; interview schedule included as supplemental file. Due to funding limitations, focus group was not audio recorded; field notes taken.	Analysis performed by two researchers (named with credentials and relationship to participants). Analysed thematically (not referenced); analytic steps not described.	Presentation of analysis is descriptive; informed by field notes so does not include participant quotes. COREQ criteria for reporting qualitative research.	Ethics approval confirmed and mention of consent for qualitative component. No attention to the influence of researcher positionality/ decisions on outcomes.	No rationale provided for mixed methods design. Described as sequential explanatory mixed-methods design.	Results of each method are presented separately with no integration. No explicit integration in Discussion.
Pines et al. (2018)	Implicit justification – study aims align with qualitative design but no explicit rationale.	Sampling/recruitment strategy not stated. Recruitment goal not stated. Non-responders/non-participants not addressed. Sample size particularly small (*n* = 5). Participant profile provided.	Unclear who conducted interviews, where, or how long they lasted. Topics summarised; not included as supplementary file. Guide piloted and revised.	Analysis performed independently by three researchers (not stated who or their credentials). Grounded theory (Glaser & Strauss, 1967) -brief summary of analytic steps.	Presentation of analysis retains contextual information (e.g. resources, patient beliefs, medical setting etc.) & supported by appropriate participant quotes. Strength of endorsement supported by % of comments related to that concept. Lack of consistency between grounded theory method and presentation of results. No reporting guidelines used.	Ethics approval confirmed and mention of consent for qualitative component. No attention to the influence of researcher positionality/ decisions on outcomes.	No rationale provided for mixed methods design. Embedded design but not explicitly stated.	Qualitative and quantitative findings are presented separately in the results section. In discussion there is brief integration of findings.
Van den Bruel (2016)	Implicit justification – study aims align with qualitative design but no explicit rationale.	Sampling strategy differed across participant groups (combined whole population and purposive sampling). No recruitment goal stated. No information given regarding composition and eventual characteristics of the small samples achieved (12 parents and 7 clinicians).	Who conducted data collection and aspects relating to practical arrangement described. Topic guides not described; not included as supplemental file.	Who performed analysis not stated. No named (or referenced) analytic approach – analytic steps poorly described. Codes and themes discussed and verified among research team and public advisory panel.	Minimal presentation of qualitative data, but clear in terms of original objectives; selected participant quotes in formatted boxes. No reporting guidelines used.	Ethics approval mentioned but no discussion of consent for qualitative aspect. No attention to anonymity and confidentiality or the influence of researcher positionality/ decisions on outcomes.	No rationale provided for mixed methods design. Qualitative study described as embedded but no explicit of this as a design.	Integration is implicitly included in the Discussion, with the qualitative findings being used to explain the quantitative findings.
Williams et al. (2008)	Implicit justification – study aims align with qualitative design but no explicit rationale. Study was time series, multiple data collection points - rationale inferred but not stated explicitly.	Sampling strategy differed across participant groups (combined whole population and convenience sampling). For patients, recruitment goal stated but not justified. Brief participant profiles provided. No non-participants or non-responders.	Who performed data collection not stated. Interviews not recorded - no rationale provided. Field notes are mentioned but insufficiently described. Topic guide contents not stated; not included as supplemental file.	Who performed analysis stated. Content analysis - no reference – analytic steps poorly described. Analysis conducted across sites and time points.	Presentation of analysis retains contextual information (e.g. resources, patient beliefs, medical setting etc.) & supported by appropriate participant quotes - although there are some long sections unsupported by quotations. Attention is given to aspects of findings that differed across time points. No reporting guidelines used.	Confirms ethics approval was granted, and participants consented. No attention to anonymity and confidentiality or the influence of researcher positionality/ decisions on outcomes.	No rationale provided for mixed methods design (authors do not refer to mixed methods design). Embedded design but also not stated.	Qualitative and quantitative findings are presented separately in the Results section. In Discussion, authors integrate findings (e.g., use qualitative findings to explain positive or surprising quantitative findings).

^i^How defensible is the research design?

^ii^How well defended is the sample design/target selection of cases/documents?

^iii^Sample composition/case inclusion – how well is the eventual coverage described?

^iv^How well was the data collection carried out?

^v^How well has the approach to, and formulation of, the analysis been conveyed?

^vi^How clear are the links between data, interpretation and conclusions – i.e. how well can the route to any conclusions be seen?

^vii^How clear and coherent is the reporting?

^viii^What evidence is there of attention to ethical issues?

#### Qualitative studies

A qualitative study design was appropriate for all qualitative studies to meet their study aims which centred on gaining a deeper understanding of experiences/attitudes towards a particular diagnostic test/technology and/or to identify barriers and facilitators to implementation. However, only five studies provide an explicit argument to justify how qualitative methods help meet these aims [15, 21, 24, 37, 41]. The others relied on an implicit justification that qualitative methods were an appropriate approach to answering research questions related to experience.

Many of the included studies sought to, and were successful in, gaining diverse experiences through opportunistic [21] or purposive sampling [15, 16, 23, 25, 35, 39, 41]; although two studies did not explicitly state what criteria/participant characteristics guided their purposive strategy [25, 35]. Some studies [24, 26, 28, 33, 37] used whole population sampling where all sites/health care professionals involved in implementation/trial were invited to participate in the qualitative sub-study and were successful in recruiting all [24, 26] or all-but-one eligible sites/participants [28, 33]. For a minority of studies, the sampling and recruitment strategies were unclear [29, 31, 36, 37].

Data collection procedures were generally well described across the 18 qualitative studies. Eleven studies described who performed the data collection along with their credentials [15, 16, 23–26, 29, 33, 35, 39, 41]. Fourteen described interview practicalities (where, when, audio recording, transcription etc.) [15, 16, 23–26, 29, 31, 33, 35–37, 39, 41], and twelve summarised the interview topic guide [16, 23–26, 31, 33, 35–37, 39, 41]. Two of the studies conducted ethnographic observations and qualitative interviews [21, 28]; only one described processes for capturing field notes/memos and conducting interviews [21].

However, the analytic approach was less consistently described; ten out of the eighteen qualitative studies failed to include a reference or else insufficiently describe the analytic steps performed [15, 16, 24, 27–29, 31, 33, 35, 36]. Most of the included studies conducted inductive qualitative analyses. Exceptions included studies which performed a deductive analytical approach informed by theoretical framework [26, 29, 39], and studies which mapped inductive analysis of data to theoretical models [28, 37]. Xie et al.’s (2024) analysis was also informed by a theoretical framework, but the process is not clearly stated [21]. None of these studies mentioned whether their analyses identified unrepresented aspects and/or inconsistencies with the guiding theoretical framework.

While this review was not concerned with the results of the included studies in terms of content, we were interested in how results were reported and the internal consistency between results and the research aims, data collection, and data analysis approaches. Most studies (*n* = 14) presented an analysis that retained pertinent contextual information (for example, professional role, patient profile, patient pathway, system logistics etc.) and interpretations/claims were well supported by participant quotes [15, 16, 21, 23–26, 28, 31, 33, 35, 37, 39, 41]. In contrast, in two studies there were discrepancies between the described analytic approach and the presentation of findings, for example, code checking being inconsistent with the analytic approach [23, 33].

With one exception, all the included studies confirmed that ethical approval had been obtained for the study and participants provided informed consent. None of the included studies attended to provided explicit details around specific components of ethical study design (e.g., confidentiality, anonymity, withdrawal, power positions etc.) or reflexivity.

#### Mixed methods studies

Of the ten included mixed methods studies, three provide an explicit justification for combining quantitative and qualitative methods [17, 30, 40]. Their justification hinges on the idea that quantitative and qualitative approaches serve to answer different questions/aims related to implementation. In two other studies, this justification was inferred [22, 38]. None of the included mixed methods studies provide an explicit justification for the qualitative component of the study.

Purposive sampling was used in two of the included studies [22, 32]; whole population sampling in five studies [14, 17, 18, 30, 40]; and mixed purposive and whole population sampling (dependent on participant group) in two studies [20, 34]. None of the included studies state a recruitment goal/target. For one study, the sampling strategy was unclear [38]. Additionally, two of the included studies do not describe the participants included in the study [22, 40].

Six studies described who performed the qualitative data collection along with their credentials [14, 17, 18, 30, 32, 40]; seven described interview practicalities (where, when, audio recording, transcription etc.) [14, 17, 22, 30, 32, 38, 40]; and six summarised the topic guide [14, 22, 30, 32, 38, 40]. In three studies there was a lack of clarity in reporting data collection [18, 20, 34].

There were omissions in the reporting of qualitative analysis in all the included mixed methods studies. Frequent omissions included, not referencing and/or sufficiently describing the analytic process [14, 17, 18, 20, 22, 30, 32, 34, 40] and not stating who performed the analysis [17, 20, 38, 40].

Most studies presented an analysis that retained pertinent contextual information (for example, professional role, patient profile, patient pathway, system logistics etc.) and interpretations/claims that were well supported by participant quotes [14, 17, 22, 30, 32, 40]. However, in four studies, the data was not organised thematically or in domains/categories but instead presented as lists and/or brief descriptions of data [18, 20, 34, 38].

Integration of quantitative and qualitative data is a core feature and strength of employing a mixed methods design. The handling and depth of integration varied across studies; in four studies there was no integration of quantitative and qualitative data [18, 20, 22, 32]; in three there was explicit integration [14, 17, 30]; and in two there was implicit integration [34, 38]. Only two studies summarised how integration was handled in the methods section [14, 40]. However, in one of these cases the integration plan did not align with the presentation of the Results or Discussion where there was no evidence of explicit integration [40].

### Use of terminology around evaluation and guiding frameworks

The studies applied various descriptive terms to articulate their objectives and outcomes (see Tables 4 and 5). All studies were interested in gaining an understanding of how the diagnostic test/technology was experienced and/or viewed by users. Terms such as ‘acceptability’, ‘acceptance’, ‘usability’, and ‘satisfaction’ were frequently used [20, 22, 25, 29–32, 34, 40], as was the explicit motivation to identify barriers/challenges and facilitators to implementation [15, 16, 19, 22, 24–29, 31, 33–36, 38, 41].

**Table 4:** Aims and Evaluative terms qualitative studies

Study	Aims	Evaluative terms used
Asiimwe (2012)	“[The] perceptions, attitudes, beliefs, and practices of these users [healthcare centre attendees and health workers] were documented and assessed to gain insight on the barriers and facilitators of acceptance and use of mRDTs [malaria rapid diagnostic test].”	Feasibility*, acceptability* & use; perceptions, attitudes, beliefs & practices, barriers & facilitators to implementation
Boadu (2016)	“To identify the factors directly influencing mRDT implementation at primary care facilities in a Ghanaian district.”	Compliance, acceptability*, perspectives & use, experiences of use, guideline knowledge and perceived challenges/advantages to implementation
Brookes-Howell (2019)	“To explore experiences of using the Flexicult test to inform management of UTI and views on requirements for an optimal POCT to inform successful implementation.”	Experiences and opinions of using Flexicult to inform management decisions, ability of Flexicult to overcome existing challenges
Cals (2010)	“To explore GPs’ attitudes to and experiences of introducing C-reactive protein (CRP) point of care testing (POCT) for lower respiratory tract infections (LRTI) in primary care.”	Attitudes, experiences & views of use (not practice audit), influence of CRP POCT on practice, barriers and opportunities to implementation (CRP uptake)
Dahm (2017)	“To gain a context-rich understanding of the operational impact of the NSW rollout of POCT across rural and remote ED settings as experienced by frontline clinical staff.”	Experiences of use, perception of POCT on work practice, improvements to POCT
Daly (2023)	“The purposes of this study were to (1) describe the characteristics of participating PBRNs and their respective collaborators in the 2322 trial, and (2) describe complications challenging its execution.”	Complications/barriers to implementation, perceptions of pros & cons of the 2322 device
Dixon (2021)	“To explore clinicians’ perspectives on having POC tests available during OOH [out-of-hours] home visits.”	Experiences & perceptions of POCT use, decision making (whether to use POCT), barriers & facilitators to implementation
Eley (2018)	“To explore the knowledge, skills, attitudes and beliefs of general practice staff about C reactive protein (CRP) point of care tests (POCTs) in routine general practice and associated barriers and facilitators to implementing it to improve the management of acute cough.”	Knowledge, skills, attitudes & beliefs about CRP POCT, barriers & facilitators to implementation, understand behavioural determinants (using COM-B), views of general practice staff who have not trialled CRP POCT
Geis (2023)	“In order to identify barriers and facilitators to the implementation of these tests, we aimed to describe GPs’ opinions of using procalcitonin and lung ultrasonography during the UltraPro clinical trial.”	Acceptance & use of POCUS, barriers & facilitators to implementation, opinions and experiences using device
Jung (2025)	“To investigate the attitudes and experiences of GPs in Germany regarding the use of semi-quantitative C-reactive protein point of care tests (CRP-POCTs).”	Implementation in routine care, understand experiences, opportunities, and barriers for use, impact on GP-patient communication, feasibility
Kornelsen (2023)	“Evaluates FPs’ [family physicians] experience and use of POCUS in their clinical practice.”	Understand experiences & perspectives about POCUS use, identify scope of POCUS practice
Rutter (2024)	“To explore the views of primary care healthcare professions and patients on the use of FebriDx to safely reduce antibiotic prescriptions for LRTIs in primary care.”	Understand views and experiences of FebriDx use, feasibility, barriers & facilitators to implementation
Shahrzad (2023)	“To explore how a device for PCR testing (Cobas® Liat®) was adopted and experienced by health professionals in general practice.”	Understand use and experiences of implementing POC PCR testing, barriers & facilitators to implementation
Simonsen (2024)	“How do GPs and staff experience and perceive Point of care diagnostic technologies?”	Understand use and experiences of implementing POC PCR testing, barriers & facilitators to implementation
Suttels (2023)	“To gain in-depth insights in the perceptions on POC-LUS and computer-assisted POC-LUS for the diagnosis of lower respiratory tract infections (LRTIs) in a low-income and middle-income country (LMIC) of sub-Saharan Africa.”	Barriers & facilitators to implementation, perceptions, explore clinical need, influence on patient dynamic & perceptions of POC-LUS
Tan (2017)	“To explore dengue surge experiences before and after POCT kit introduction so as to elicit recommendations to improve quality of care delivery for dengue patients in Singapore and in similar high-income endemic settings.”	Understand patient journey†, narrative approach to elicit experiences, perspective of patient and health care professional, synthesise findings to improve model of care delivery
Xie (2024)	“To explore factors that influence the adoption or non-adoption of POCT in English general practices and provide insights to enable its integration into routine practice workflows.”	Ethnographic observation, explore use & perceptions of RTI POCT
Zongo (2018)	“To analyze the use of rapid diagnostic tests in six health and social promotion centers (i.e. health-care centers, from the French Centers de Santé et de Promotion Sociale) of Ouagadougou (Burkina Faso) in an exploratory research context.”	Use & perceptions of mRDT, prescribing practices, challenges/issues with implementation

*Uses a theoretical framework to define and operationalise acceptability and use of diagnostic tests (learnability, willingness, suitability, satisfaction, efficacy, effectiveness)

†Informed by patient-centred Process Mapping approach which operationalises patient experience as symptom experience & help seeking, receiving diagnosis, treatment & recovery – pathway mirrored for clinician perspective as patient arrival, giving diagnoses, treatment & discharge

**Table 5:** Aims and Evaluative terms mixed methods studies

Study	Aims	Evaluative terms used
Bennis (2024)	“To investigate the micro-costing and acceptability of Cutaneous Leishmaniasis diagnostic tools in Morocco from the perspective of healthcare professionals.”	Economic evaluation (micro-costing), acceptability & experience of CL-RDT use
Blattner (2019)	“To describe the impact of the introduction of POC haematology at Hokianga Hospital on patient care, the acceptability to medical and nursing staff and a cost–benefit analysis.”	Impact, acceptability, cost-benefit analysis
Boere (2022)	“The aim of this process evaluation is (1) to determine intervention quality regarding CRP POCT utilization in the UPCARE study, and (2) to determine barriers and facilitators for the implementation and utilization of CRP POCT in the NH setting.”	Logistics & experiences of use, barriers & facilitators, CRP utilisation & appraisal, intervention quality; experiences related to satisfaction, relevance, feasibility & fidelity
De Vos (2023)	“To assess the acceptability and usability of a novel lateral flow assay and portable reader for the point of care detection of N. gonorrhoeae infection (NG-LFA).”	Device development & feasibility, acceptability*, usability*, implications for implementation, experiences & preferences for scale-up
Dixon (2022)	“We conducted a mixed methods evaluation of this service improvement [CRP POCT offered in out-of-hours assessment bases].”	Service improvement evaluation, experiences of CRP POCT (non)use
Hayward (2020)	“To evaluate test usage and patient and clinician experience following the introduction of point of care (POC) blood tests into a primary care out-of-hours service.”	Service improvement evaluation, clinician & patient views, describe usage
Hodge (2025)	“The primary objective of the study was to understand the experiences of specialist paramedics using POCT devices in pre-hospital urgent and emergency care settings, along with their self-reported impact on clinical decision making. The secondary objectives were to establish the practicality of using POCT devices in this setting and to understand in which patient presentations the device is most used.”	Feasibility evaluation, understand experience using POCT, self-reported impact on decision making, describe usage
Pines (2018)	“To examine the impact of POCT in a single, community ED.”	Impact of intervention on time to test lab results and length of stay, staff report of use, perspectives on impact, perceived benefits & challenges to use
Van den Bruel (2016)	“To determine the acceptability and impact of CRP testing in acutely ill children.”	Feasibility, acceptability, uptake, parent & clinician perspectives, utility/clinical value
Williams (2008)	“To evaluate the impact of RDTs on prescribing behaviours, assess prescribers’ and patients’ perceptions, and identify operational issues during implementation.”	Impact (on prescribing behaviour), usage, patient & clinician perceptions on use of RDTs, identify operational issues, acceptance & satisfaction

*Used theoretical framework (Health Technology Adoption Framework) to define feasibility (i.e., acceptance & usability define feasibility) – domains further divided into learnability, willingness, satisfaction, suitability, efficacy, and effectiveness

However, only four studies defined the evaluative terms they used [29, 31, 32, 39]. Of these, three used the Health Technology Adoption Framework which had pre-defined terms that were adapted by the authors to suit their study context [29, 31, 39]. For example, de Vos (2023) omitted sub-domains ‘willingness’, ‘efficacy’, and ‘effectiveness’ arguing with some justification that they were not relevant to the study objectives and feasibility was not being investigated [32]. Additionally, de Vos (2023) added the sub-domain ‘durability’ to their model to capture aspects related to healthcare systems/challenges unique to low-resource settings [32]. Furthermore, adapting the model to respond to the research context resulted in the same terms being re-defined, resulting in different definitions across studies. For example, although the term ‘learnability’ was used in two studies [29, 32], in one study the term related to confidence, training and easy/difficult learning process [32], while in the other it related to the ability of the healthcare worker to use the test [29]. One study aligned its work with the patient-centred Process Mapping approach [39]. The authors used this framework not to elucidate the definitions of terms, but to operationalise patient (and clinician) experience, thereby scaffolding the study objectives and outcomes.

## Discussion

### Summary

This systematic review identified 28 studies using qualitative and mixed methods approaches in the evaluation and implementation of diagnostic health technologies. All the studies included in this review concerned evaluation and implementation of point of care tests or technologies, many of which were conducted in primary care/general practice in the UK or Europe to diagnose respiratory infections. Just under two thirds (*n* = 18) employed a qualitative only design and around one third (*n* = 10) a mixed methods design. Most studies used semi-structured interviews conducted with healthcare professionals as either the sole or principal data collection method.

### Considerations regarding quality and reporting

Across the studies identified for inclusion, there were a number of issues related to quality and reporting, which offer learning opportunities for future research. In order to generate important insights and evidence around the “wider impacts” of diagnostic testing and technology acceptable to commissioning and policy-making bodies, qualitative and mixed methods studies need to be sufficiently grounded in, and engaged with, the relevant methodological and/or research design literatures, especially where access to relevant methodologists or methodological support may be limited. A number of the studies were not explicit in their reporting about their rationale for the approaches used and we consider this to be a key omission, as such information would be helpful for others planning similar evaluations. Where qualitative methods are used (particularly semi-structured interviews, the most common qualitative method reported in the included studies), researchers could explain the increased understanding offered by qualitative approaches when exploring and making sense of people’s experiences. In mixed methods studies, researchers could be more explicit in explaining the extra value which can be derived from combining qualitative and quantitative methods, for example how it allows them to more comprehensively assess the technology. They could be more attentive to the sequence, timing and integration of the quantitative and qualitative components which are central tenets of robust mixed methods design. [42]

Similarly, it is important to acknowledge the non-equivalence of certain methods. In some of the included studies, individual interviews and focus groups did not appear to be viewed as distinct ways of generating data. We would argue that focus groups should not be regarded as a convenient way of capturing the perspectives of multiple participants simultaneously but recognised as a distinct qualitative method which is able to respond to certain types of research question, for example, where a discussion-based approach to understanding issues which would be important in shaping implementation into real-life settings is required.[43]

In aiming to generate evidence of “wider impacts” and provide the valuable insights into diagnostic testing and technology, researchers could also consider the range of methods at their disposal and avoid reliance on one method, such as individual interviews, with mainly professional stakeholders. The methodological literature contains examples of emerging creative and innovative qualitative methods, which offer a variety of ways to capture and potentially improve patient experiences, including documentary (e.g. where researchers could use diaries to collect data on the feasibility and acceptability of tests/technologies to patients) and participatory approaches (e.g. where researchers and patient/public stakeholders collaborate to make the design/delivery of tests/technology more accessible and acceptable) [8], as well as a growing body of research involving more established methodologies such as ethnography and focused ethnography.[44, 45]

Across the majority of the included studies, there was a lack of, or insufficient, transparency in data analysis, ranging from the omission of methodological referencing to the lack of detail around the analytic steps taken to produce the reported results. We acknowledge that it is a challenge, for mixed methods studies especially, to describe all pertinent study details within the parameters of journal word limits. Making available more information about methodology as supplementary material could be a solution to this. The majority of the studies also did not choose to use existing reporting checklists (see Tables 2 and 3). These can support the provision of more relevant and useful methodological information and we would encourage more journals to recommend use of these as part of publication. Published articles are often used to ground and guide researchers in the current accepted approaches for conducting research and the conventions to report their work. This is one of the reasons why, in a developing field area of interest, such as diagnostic testing and technology, clear and transparent reporting is of particular importance.

### Considerations regarding terminology and frameworks

Many of the papers included in this review characterised the qualitative and mixed methods approaches they used as a means of exploring the experiences and perceptions of the test/technology users (see Tables 4 and 5). In this respect, we frequently encountered terms such as ‘usability’, ‘acceptability’ and ‘feasibility’ of the tests/technology, but found these were used idiosyncratically and without definitions. Consequently, under these and similar terms, different information was captured in different papers. This is a similar finding to Sekhon et al. (2017) which explored how ‘acceptability’ was defined and assessed as part of the design and evaluation of healthcare interventions and called for research into ‘acceptability’ to be more robust in this respect. [46] Providing definitions of terms used so that others do not have to infer what they mean could be a useful consideration when reporting research.

Only a small minority of the papers we reviewed employed theoretical models/frameworks around adoption and implementation. Where they did, some of the studies drew on transparent definitions of the aspects around which they then constructed their research design. We also encountered studies where the full model was not used, and definitions were not applied in the expected way. It appeared that the use of appropriate and relevant frameworks can offer researchers help in clarifying evaluation questions which qualitative and mixed methods research can then answer. This may be of particular help to less experienced researchers but is not restricted to this group.

### Considerations regarding patient perspectives

Overall, the included studies lacked both PPIE and exploration of patient perspectives, even where these would have been pertinent and could have added value to the evaluations. We are aware that the experiences of the testing process for patients who participate in some evaluations of diagnostic tests would be the same as if they were receiving usual care. For example, in diagnostic accuracy studies where samples are taken in the same way (through capillary blood sample, urine sample etc.) but the index test(s) will only be compared with the reference standard to determine accuracy and do not have any bearing on the decision-making around diagnosis for the individual who has consented to take part in the study. However, gaining patient views on how tests might be included in future care could be helpful. In studies where patients did experience changes in the testing process, the lack of patient views is harder to explain. It may be that approval for exploring staff views was an accepted component of the evaluation in some of the studies and exploring the views of patients would have required additional ethics approvals, which required more time and resources. It is also possible that among researchers in this field ‘diagnostic testing’ is viewed as concerning clinicians more than patients, as it is clinicians who determine what testing, if any, is necessary for diagnosis in individual patients, and patient outcomes are downstream of decision-making around diagnosis. However, capturing broader impacts of new diagnostic tests and technology seems vital, as it is it is always likely to be the case that patient care will be affected by them and the investment of the necessary resources in qualitative and mixed methods research would be worthwhile.

### Strengths and limitations

Within the rapidly expanding field of diagnostic health and technologies, there have been a number of systematic reviews of methods for evaluation but these have focused on quantitative and health economic aspects [47, 48, 49]. To the best of our knowledge, this is the first systematic review undertaken to explore qualitative and mixed methods approaches to evaluation in this field. This paper presents and critiques the studies we identified and offers some pointers for enhancing the quality, breadth and reporting of the approaches undertaken.

While our search resulted in studies across a wide range of conditions and settings, our main criteria for inclusion were that papers focused on the evaluation or implementation of particular tests/technology and that qualitative and/or mixed methods research were used and reported. Not meeting these inclusion criteria accounted for the majority of papers we rejected.

A limitation of this review, as a result of our narrow focus, is that among the papers which met our inclusion criteria, the focus was on point of care diagnostics. Thus, we were unable to capture the qualitative and mixed methods approaches which may have been employed for evaluations of other types of diagnostics or health technology. Additionally, we did not seek additional clarification on the rationale or methods from study authors where this was not reported, meaning that we have not described decisions or processes which were not included in the published manuscript.

### Implications for research and practice

Our review highlights that in many cases quantitative methods alone are used for the evaluation of new diagnostic technology. Thus “wider aspects” often remain unexplored, with any qualitative and mixed methods approaches either not included in the evaluation or remaining unpublished. Our review also shows that where qualitative and mixed methods research approaches are used in the evaluation and implementation of new diagnostic technology, there are issues around methodological quality and reporting, as well as the use of terminology and guiding frameworks.

While methodological guidance to support the adoption of qualitative and mixed methods has emerged in the evaluation of health care and public health interventions [7, 8, 50, 51], there is still a lack of methodological guidance which could support their adoption in this developing field. Our review also suggests where evaluation is being done, the field would benefit from clearer guidance to support choice and justification of method, definitions of terminology used, alongside improved reporting of conduct and analysis of qualitative and mixed methods approaches, to ensure the generation of findings which can support implementation and adoption. While generic appraisal tools, such as the ones used in this review, could provide important indicators of good practice in the conduct and reporting of qualitative and mixed methods research, some form of consolidated guidance, developed using consensus methods, to support the use of these methodological approaches in this field, including information around relevant published frameworks, would be timely and useful. This could supplement the guidance which already exists to support the required reporting of quantitative studies on the diagnostic accuracy of tests and technologies [52].

It could be argued that any attempt to include exploration of wider impacts around diagnostic technology would be better than not exploring them at all, especially where resources for this are lacking. However, for evidence from research to be seriously considered, a certain level of rigour would be expected or required. The development of a guiding framework could take this dilemma into account, for example by setting out minimum essential design and reporting requirements for qualitative and mixed methods in diagnostic evaluations and less essential but desirable characteristics. As diagnostic technologies are set to proliferate, higher quality, more standardised approaches which can count as evidence of “wider impacts” in formal assessment programmes such as NICE and in regulatory processes are required and an evidence-based guiding framework could help to meet this need.

## Electronic supplementary material

Below is the link to the electronic supplementary material.

Supplementary Material 1

## Data Availability

All raw data collected about each study and appraisal of methods used is available within the manuscript (Tables 1-5).
