# Supplementary material for: Use of qualitative and mixed methods research in diagnostic technology evaluation and implementation: insights from a systematic review
Source: Diagn Progn Res. 2026 Oct 7;10:26. doi: 10.1186/s41512-026-00240-z (PMC13644275; doi:10.1186/s41512-026-00240-z)
Supplement: Supplementary file 1 [file 41512_2026_240_MOESM1_ESM.docx]

| 1 | Primary Health Care/ |
| --- | --- |
| 2 | exp General Practice/ |
| 3 | general practitioners/ or physicians, family/ or physicians, primary care/ |
| 4 | Office Visits/ |
| 5 | House Calls/ |
| 6 | exp Ambulatory Care Facilities/ |
| 7 | Emergency Service, Hospital/ |
| 8 | exp Emergency Medicine/ |
| 9 | Community Health Nursing/ or Nurses, Community Health/ |
| 10 | (primary adj (care or healthcare or health care)).ti,ab,kf. |
| 11 | ((general or family) adj2 (practi* or physician? or doctor?)).ti,ab,kf. |
| 12 | (gp or gps).ti,ab,kf. |
| 13 | (health centre? or health center? or medical centre? or medical center? or clinic?).ti,ab,kf. |
| 14 | ((house or home) adj (call? or visit?)).ti,ab,kf. |
| 15 | (ambulatory adj2 (care or health* or center? or centre?)).ti,ab,kf. |
| 16 | ((accident adj2 emergency) or (emergency adj (department? or dept? or ward? or unit?))).ti,ab,kf. |
| 17 | (((community or family or home) adj2 nurs*) or district nurs*).ti,ab,kf. |
| 18 | 1 or 2 or 3 or 4 or 5 or 6 or 7 or 8 or 9 or 10 or 11 or 12 or 13 or 14 or 15 or 16 or 17 |
| 19 | Diagnostic Techniques and Procedures/ |
| 20 | Diagnostic Tests, Routine/ |
| 21 | exp Point-of-Care Systems/ |
| 22 | exp Hematologic Tests/ |
| 23 | Urinalysis/ |
| 24 | Saliva/an [Analysis] |
| 25 | exp Blood/an [Analysis] |
| 26 | Urine/an [Analysis] |
| 27 | exp Breath Tests/ |
| 28 | ((diagnostic or invitro or "in vitro") adj2 (test* or technolog* or technique?)).ti,ab,kf. |
| 29 | ((("point of care" or POC or rapid or "near patient" or bedside or bed-side) adj2 (test* or diagnos* or ultraso* or sonogra*)) or poct or pocus).ti,ab,kf. |
| 30 | ((h?ematolog* or blood) adj2 test*).ti,ab,kf. |
| 31 | (finger prick* or fingerprick*).ti,ab,kf. |
| 32 | ((urin* adj2 test*) or urinalysis).ti,ab,kf. |
| 33 | (saliva* adj2 test*).ti,ab,kf. |
| 34 | (breath adj2 test*).ti,ab,kf. |
| 35 | 19 or 20 or 21 or 22 or 23 or 24 or 25 or 26 or 27 or 28 or 29 or 30 or 31 or 32 or 33 or 34 |
| 36 | exp "diffusion of innovation"/ |
| 37 | device approval/ or diagnostic test approval/ |
| 38 | Program Evaluation/ |
| 39 | practice patterns, nurses'/ or practice patterns, physicians'/ |
| 40 | Health Knowledge, Attitudes, Practice/ |
| 41 | (implementation or adoption or ((test* or technolog* or innovat* or diagnostic?) adj5 (implement* or adopt* or transfer*))).ti,ab,kf. |
| 42 | (barrier? or obstacle? or challenge? or facilitator? or enabler? or opportunit*).ti,ab,kf. |
| 43 | (utili?ation or utility or usage).ti. or ((test* or technolog* or diagnostic?) adj5 (utili* or usage)).ti,ab,kf. |
| 44 | (acceptability or usability or human factor?).ti,ab,kf. |
| 45 | 36 or 37 or 38 or 39 or 40 or 41 or 42 or 43 or 44 |
| 46 | grounded theory/ or exp qualitative research/ |
| 47 | focus groups/ or interviews as topic/ or "surveys and questionnaires"/ |
| 48 | qualitative.ti. or (qualitative adj2 (research or stud* or evaluat*)).ti,ab,kf. |
| 49 | (interview* or focus group? or survey? or questionnaire?).ti,ab,kf. |
| 50 | (grounded theory or ipa or phenomenolog* or hermeneutic? or ethnograph* or thematic analys* or conversation analys*).ti,ab,kf. |
| 51 | ((mixed or multi*) adj2 method*).ti,ab,kf. |
| 52 | 46 or 47 or 48 or 49 or 50 or 51 |
| 53 | 18 and 35 and 45 and 52 |
